# An Increase in Consuming Adequately Iodized Salt May Not Be Enough to Rectify Iodine Deficiency in Pregnancy in an Iodine-Sufficient Area of China

**DOI:** 10.3390/ijerph14020206

**Published:** 2017-02-20

**Authors:** Zhifang Wang, Wenming Zhu, Zhe Mo, Yuanyang Wang, Guangming Mao, Xiaofeng Wang, Xiaoming Lou

**Affiliations:** 1 Department of Environmental and Occupational Health, Zhejiang Provincial Center for Disease Control and Prevention, 3399 Binsheng Road, Hangzhou 310051, China; zfwang@cdc.zj.cn (Z.W.); wmzhu@cdc.zj.cn (W.Z.); zhmo@cdc.zj.cn (Z.M.); yywang@cdc.zj.cn (Y.W.); 2 Key Medical Research Center, Zhejiang Provincial Center for Disease Control and Prevention, 3399 Binsheng Road, Hangzhou 310051, China

**Keywords:** iodine, iodine deficiency, urinary iodine concentration, pregnancy

## Abstract

Universal salt iodization (USI) has been implemented for two decades in China. It is crucial to periodically monitor iodine status in the most vulnerable population, such as pregnant women. A cross-sectional study was carried out in an evidence-proved iodine-sufficient province to evaluate iodine intake in pregnancy. According to the WHO/UNICEF/ICCIDD recommendation criteria of adequate iodine intake in pregnancy (150–249 µg/L), the median urinary iodine concentration (UIC) of the total 8159 recruited pregnant women was 147.5 µg/L, which indicated pregnant women had iodine deficiency at the province level. Overall, 51.0% of the total study participants had iodine deficiency with a UIC < 150 µg/L and only 32.9% of them had adequate iodine. Participants living in coastal areas had iodine deficiency with a median UIC of 130.1 µg/L, while those in inland areas had marginally adequate iodine intake with a median UIC of 158.1 µg/L (*p* < 0.001). Among the total study participants, 450 pregnant women consuming non-iodized salt had mild-moderate iodine deficiency with a median UIC of 99.6 µg/L; 7363 pregnant women consuming adequately iodized salt had a lightly statistically higher median UIC of 151.9 µg/L, compared with the recommended adequate level by the WHO/UNICEF/ICCIDD (*p* < 0.001). Consuming adequately iodized salt seemed to lightly increase the median UIC level, but it may not be enough to correct iodine nutrition status to an optimum level as recommended by the WHO/UNICEF/ICCIDD. We therefore suggest that, besides strengthening USI policy, additional interventive measure may be needed to improve iodine intake in pregnancy.

## 1. Introduction

Iodine is an essential micronutrient for human beings. Inadequate iodine intake can cause iodine deficiency disorders (IDDs), which have adverse effects on quality of human life. Iodine deficiency can irreversibly damage brain development in the fetus, infant, and child [1,2], leading to mental retardation, congenital abnormalities, and abnormal neuronal development. 

IDD is a serious public health problem in China. In the 1990s, IDD existed in all of 31 provinces (autonomous regions, municipalities) and Xinjiang Production and Construction Corps throughout the country (no data available in special administrated regions) due to insufficient environmental iodine exposure [3,4]. At least 425 million people were estimated to be at risk of IDD, including 7 million endemic goiters and 8.25 million endemic cretinism cases. It accounted for 40% of the global total [5,6]. To control, prevent, and eliminate IDD, China first introduced iodized salt in 1995 and implemented a universal salt iodization (USI) policy via edible salt monopoly regulation, which means the State Council of China is the only official organization to set salt iodine content. 

For achieving the goal of eliminating IDD, the content of iodine in salt is critical: too little, iodized salt is ineffective as an interventive mean; too much, it can cause the targeted population to become toxic. Iodine content was initially set as 20–60 ppm at the household level between 1995 and 1996 [7]. Later, iodine concentration in salt was modified twice based on the national epidemiological surveillances results on the median urinary iodine concentration (UIC) in school-age children (SAC), which is the assessment method of iodine intake and iodine nutrition status in the population, recommended by the World Health Organization (WHO)/the United Nations Children’s Fund (UNICEF)/the International Council for the Control of Iodine Deficiency Disorders (ICCIDD) [8]. First, iodine content in salt was adjusted to 20–50 ppm in 2000 [9], when the national survey showed that the median of UIC in SAC was 306.0 μg/L in 1999, which indicated that iodine intake was excessive and iodine nutrition posed harmful risks in the overall population according to assessment criteria recommended by the WHO/UICEF/ICCIDD [8]. Second, salt iodine content was then modified to 20–30 ppm in 2012 [10] because iodine intake between 2005 and 2011 was assessed to be above the requirements [11,12] and iodine nutrition may pose a slight risk in the general population according to the WHO/UNICEF/ICCIDD recommendation criteria. China achieved the goal of eliminating IDD at the country level by 2005. However, with the country’s large size, concerns were raised that iodine nutrition were differently distributed across the region throughout the country. Therefore, the Chinese government abandoned the “one standard fits all size” and let 31 provincial governments choose their own salt iodine contents, which is accordant with iodine nutrition in the population. 

Zhejiang, a coastal province located in the eastern China, set salt iodine content with 25 ppm (Range: 18–33 ppm) in 2012 based on the national surveys results in SAC, showing more than adequate iodine intake in the population in 2011 [13]. A previous adjustment of salt iodine content was made on the basis of iodine intake and iodine nutrition in SAC, who is believed to represent the general population [8]. However, studies in pregnant women, the most vulnerable population to IDD, were scarce. Previous research in pregnant women was conducted with limited samples, and the results were not consistent. The national survey performed in 2011 showed that iodine nutrition in 456 pregnant women was adequate in the Zhejiang province [13], while epidemiological investigation in 450 and 620 pregnant women conducted by Mao et al. and Mo et al., respectively, showed that iodine nutrition in pregnancy in the province was insufficient during the same period [14,15]. Therefore, we performed a clinic-based cross-sectional study to determine iodine nutrition in pregnant women via stratified multistage sampling to represent the targeted population.

## 2. Materials and Methods

### 2.1. Sampling Design and Sample Collection 

**Figure 1:**
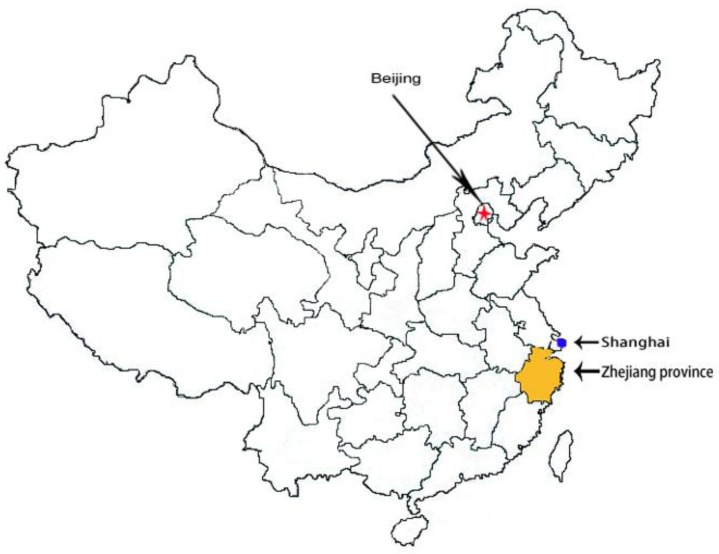
Location of Zhejiang Province, China.

Due to the large area and population of China, the administrative divisions of China consist of three levels: the provincial (province, autonomous region, municipality, special administrated region, and Xinjiang Production and Construction Corps). Zhejiang province, an area of 101,800 square kilometers, is divided into 11 city-level divisions and the 11 city-level divisions are subdivided into 90 county-level divisions along the East China Sea (Figure 1). A clinic-based, cross-sectional survey was conducted in the Zhejiang province between April and September 2015. According to the guidelines to assess the IDD national program recommended by the WHO/UNICEF/ICCIDD [8] and the national IDD surveillance protocol [16], stratified multistage sampling was performed in this study. First, each of the 11 cities in Zhejiang is selected. Second, for each selected city, all counties were selected. Third, for each selected county, five towns were randomly selected from five different locations (east, west, south, north, and the center). Finally, for each selected town, 20 pregnant women residing in the selected town for at least 6 months were randomly selected. 

For each participant enrolled, approximately 20 mL of spot urine sample was collected and sealed in a polypropylene tube with a screw top; approximately 50 g of household table salt was also collected in a plastic bag. Spot urine samples were immediately stored in a refrigerator at 4 °C. Household table salt samples were kept in a dark place at room temperature. 

### 2.2. Determination of Iodine Concentration and Iodine Status Assessment Criteria

The iodine content of household table salt was measured using iodometric direct titration. Non-iodized salt is defined as salt iodine content <5 ppm. Adequately iodized salt is categorized as iodine content of 18–33 ppm. UIC was determined by the arsenic–cerium catalytic spectrophotometry. Iodine levels of all samples were examined in the National Reference Laboratories at the county level. In this study, we adopted the assessment criteria recommended by the WHO/UNICEF/ICCIDD [8,17] (Table 1). 

### 2.3. Statistical Analysis

Data were input in Microsoft Office Excel 2007 and analyzed through SPSS (version 23.0, IBM Corporation, Chicago, IL, USA). Count data (the number of sample) were expressed as number and percentage (%). Comparisons of count data were made using the chi-square test. The non-normally distributed UIC were expressed as median and interquartile range. The distribution of UIC was described as the frequency. Frequency describes the number of total study pregnant women whose UIC fall into each of several categories (per 50 µg/L interval). The median UICs between the two groups were compared using non-parametric Mann–Whitney test. *p*-values < 0.05 were considered significant. 

Percentage (P_a_) of pregnant women consuming adequately iodized salt was calculated by dividing the number (N_18–33_) of the total study population of pregnant women eating salt containing 18–33 ppm iodine ion by the number (N_≥5_) of pregnant women using salt containing ≥5 ppm iodine (P_a_ = N_18–33_/N_≥5_).

Percentage (P_n_) of pregnant women consuming non-iodized salt was calculated by dividing the number (N_<5_) of pregnant women consuming salt containing <5 ppm iodide ions by the number (N) of all participants investigated (P_n_ = N_<5_/N). 

All 11 study cities were divided into two areas: the coast and the inland. This study analysis was based on the assumption that inhabitants living in the coastal areas more frequently consume iodine-rich foods, such as kelp and seafood, compared with those living in inland. Those cities with a long coastline, such as Jiaxing, Zhoushan, Ningbo, Taizhou, and Wenzhou, were defined as the coastal areas. The other remaining six cities, including Hangzhou, Huzhou, Shaoxing, Jinhua, Quzhou, and Lishui, were defined as the inland areas.

### 2.4. Ethical Stament

All subjects gave their informed consent for inclusion before they participated in the study. The study was conducted in accordance with the Declaration of Helsinki, and the protocol was approved by the Ethics Committee of Zhejiang Provincial Center for Disease Control and Prevention (ZJ20151222). 

## 3. Results

### 3.1. Characteristics of the Total Study Population of Pregnant Women

A total of 8518 pregnant women were selected in Zhejiang in this study. Out of the total study population of pregnant women, 95.8% (8159) of participants handed in both urine samples and household table salt samples. The mean age of the pregnant women was 28.3 years, with a standard deviation of 5.4 years. Age and geographical distribution of the total study participants are shown in Table 2. 

### 3.2. Median UIC in Pregnant Women

**Figure 2:**
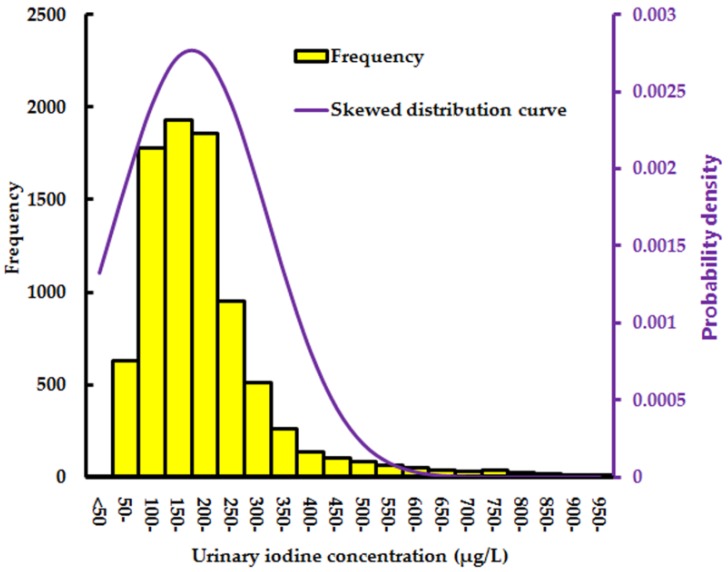
Distribution of UIC in 8510 pregnant women in the Zhejiang province.

Figure 2 showed the frequency distribution of UIC in the total study pregnant women in Zhejiang in 2015. Overall, the median UIC of the total study population of pregnant women was 147.5 µg/L (Interquartile range: 93–210 µg/L) at the provincial level. The frequency of UIC during pregnancy was a positive skewness distribution. 

The median UICs of the entire study population of pregnant women by maternal age, consuming salt, and areas are shown in Table 3. For the total study population of pregnant women, participants consuming adequately iodized salt had a statistically significantly higher median UIC of 151.9 µg/L compared with a median UIC of 99.6 µg/L in participants using non-iodized salt (*p* < 0.001). The median UIC (158.1 µg/L) of participants living in the inlands was statistically significantly greater than the median UIC (130.1 µg/L) in the coastal areas (*p* < 0.001). Moreover, for participants living in the coastal areas, those who consumed adequately iodized salt had a significantly higher median UIC (135.8 µg/L), compared with those using non-iodized salt, who had a median UIC of 114.0 µg/L (Z = −6.280, *p* < 0.001). For pregnant women in the inland areas, those who consumed adequately iodized salt had a statistically higher median UIC (159.0 µg/L), compared with those using non-iodized salt, who had a median UIC of 145.1 µg/L (Z = −2.520, *p* < 0.05).

In addition, the median UIC of pregnant women was not statistically significantly different between two age groups (*p* > 0.05), with a median UIC of 149.9 µg/L in participants aged ≤30 years old and 143.9 µg/L in those >31 years, respectively.

### 3.3. Distribution of Salt Containing Different Iodine Content

Of 8159 salt samples, 450 (5.5%) samples were determined as non-iodized salt (<5 ppm iodine level in salt), 7709 (94.5%) as iodized salt, and 7355 (90.1%) as adequately iodized salt (18–33 ppm). In addition, 354 (4.3%) salt samples were measured as non-adequately iodized salt, with 291 (3.6%) samples containing 5–18 ppm iodine content and 63 (0.8%) samples having ≥34 ppm, respectively. 

Table 4 shows the distribution of non-iodized salt and adequately iodized salt consumed by the total study population of pregnant women by gestational age and areas in 2015 in the Zhejiang province. The percentage of participants consuming adequately iodized salt was not statistically significantly different between the two age groups, with 94.5% for the group ≤30 years and 93.5% for >31 years, respectively (*p* > 0.05). Adequately iodized salt was consumed among approximately 90% of the participants in the coastal areas and among approximately 99% of the participants living in inland areas, which was a statistically significant difference between the coastal and the inland areas (*p* < 0.001).

### 3.4. Distribution of UIC in Pregnant Women Consuming Salt Containing Different Iodine Content

Among the entire study population (8510 participants), 51.0% of them had a UIC < 150 µg/L, 32.9% had a UIC of 150–249 µg/L, 12.7% had a UIC of 250–499 µg/L, and 3.3% had a UIC ≥ 500 µg/L. For those consuming adequately iodized salt, 49.5% of them had a UIC < 150 µg/L, and 33.8% had a UIC of 150–249 µg/L. Compared with the percentages of UICs in pregnant women consuming adequately iodized salt, a higher ratio (71.6%) of UIC < 150 µg/L and lower percentage (16.7%) of UIC of 150–249 µg/L occurred in the study population consuming non-iodized salt (Table 5). 

## 4. Discussion

To the best of our knowledge, this is the first population-based investigation on iodine intake and iodine nutrition in pregnancy in the Zhejiang province, China, covering 8159 pregnant women in 2015. 

Compared with iodine intake in the general population, pregnant women require more iodine intake due to physiological changes in iodine metabolism [18], including an increased renal clearance of iodide and an additional iodine requirement for the fetus and mother to synthesize thyroid hormone to maintain euthyroidism. According to the WHO/UNICEF/ICCIDD recommendation, pregnant women have an iodine intake requirement of 250 µg per day, while the general population require 150 µg/L per day [8]. Increased concerns on iodine nutrition were shifted from SAC to pregnant women because increased iodine intake in pregnancy makes pregnant women more sensitive to IDD [8]. In this study, we found that the median UIC in the total study population of pregnant women was 147.5 µg/L, which falls below the recommended lower cut-off level (150 µg/L) according to the WHO/UNICEF/ICCIDD criteria and denotes insufficient iodine intake during pregnancy. This indicated that a marginal iodine deficiency occurred in the pregnant woman population in the Zhejiang province, which was consistently believed to be an iodine-sufficient area during the recent decade [14,15,19,20,21,22]. The contradictive phenomenon on iodine nutrition was mainly attributed to the representative sampling from the two different populations. The previous national epidemiological investigations on iodine nutrition status preferred the SAC group because it is easily accessible via school surveys [13]. Moreover, because schoolchildren eat diets similar to the remaining people in their household, the median UIC in schoolchildren are assumed to represent iodine intake characteristics of the overall population (pregnant women included) [8]. The current result of the present study revealed that discrepancies occurred between the median UIC in schoolchildren and pregnant women in an iodine-sufficient area. These discrepancies observed were in agreement with recent studies conducted in Denmark [23], Sweden [24], and Belgium [25]. These studies indicated that the iodine status assessment in schoolchildren based on school surveys may not be appropriate to represent iodine status in the pregnant population. The assessment of iodine nutrition in pregnant women should be strengthened. However, several confounding factors were not included in the above studies, such as location and time of spot urine sampling. A recent investigation of Danish pregnant women reported that, after participants handed in a spot urine sample both in the hospital and at home, samples in the hospital were determined to have a lower median UIC than the samples at home [26]. In the same study on iodine intake in pregnancy in Denmark, no significant difference in the median UIC between pregnant women and their children was observed when they were instructed to provide spot urine samples at home at the same time. Therefore, more studies are needed to clarify these influencing factors and should be performed to emphasize the importance of regularly monitoring iodine status in the pregnant population [27,28,29]. 

Compared with previous studies that showed sufficient iodine intake in pregnancy in the Zhejiang province before adopting the new salt iodine content standard (18–33 ppm iodine inedible salt) [13,30], the present result was different once this 18–33 ppm standard was adopted. For pregnant women, previous adequate iodine status has been transformed to currently inadequate iodine status since the advent of the 18–33 ppm standard, which is in line with other studies conducted in China [31]. Chen et al. and Wang et al. observed that iodine deficiency in the pregnant population occurred after the new standard was implemented while they were previously iodine-sufficient [32,33]. However, Yang et al. reported that the sufficient iodine status in pregnant women did not change before and after adopting the new standard [34]. This difference may be explained as follows: (1) Iodine nutrition status in the population is closely related with salt iodine content. After the new standard was implemented, the changing iodine status in pregnancy from sufficiency to deficiency was observed in iodine-sufficient areas, although no difference was seen in pregnant women with sufficient status in areas with more-than-adequate iodine; (2) Because the median UIC in schoolchildren is recommended to evaluate iodine status in the general population, the authorities possibly adjusted the salt iodine content based on the surveillance results of iodine status assessment only in schoolchildren. However, pregnant women increased requirements of iodine intake, compared with the general population. It appears that iodine status in pregnant women was severely overlooked when the new salt iodine content standard (18–33 ppm iodine inedible salt) was adopted. Therefore, an alternative standard of salt iodine content should be recommended and encouraged, and an optimal iodine content of salt should be used in pregnant women. 

There are no cut-off values for distinguishing among mild, moderate, and severe iodine deficiency during pregnancy, according to the recommendation by the WHO/UNICEF/ICCIDD criteria. Based on the published literature [35,36,37,38,39], the degree of iodine deficiency in pregnancy in this study is defined as mild. It is well established that severe iodine deficiency in the uterus can cause severe and irreversible impairment in children’s cognitive development, such as cretinism. However, there are limited studies on the association between maternal mild iodine deficiency and their offspring’s subtle brain development. No randomly controlled clinical trial has measured short-term and long-term outcomes on offspring brain development. Several cross-sectional studies described marginal iodine deficiency in the uterus that had negative effects on fetal brain development [40,41,42]. A recent cohort study conducted by Hynes et al. found that mild iodine deficiency during gestation can have long-term adverse impacts on fetal cognitive development and intellectual function [43]. Therefore, we suggest that the healthcare system should pay more attention to iodine deficiency in pregnancy in areas where iodine deficiency has been eliminated.

On a worldwide basis, the general population usually obtains the necessary levels of iodine from their daily diet. In 1993, WHO and UNICEF recommended USI as the main strategy to control and eliminate IDD [44]. Until 2011, 148 countries have adopted mandatory iodization of edible salt to eliminate IDD [45]. Besides the implementation of USI policy, some countries where IDD exists also recommend that pregnant women have iodine-containing supplements. For example, after the implementation of mandatory iodine fortification in bread in 2009, the Australian National Health and medical Research Council also recommends that all pregnant women take an iodine supplement of 150 µg per day [46]. WHO and UNICEF recommend pregnant women to take iodine supplementation only in countries where lower coverage (<20%) of iodized salt occurs at the household level [47]. However, there was no recommendation for pregnant women in China when research showed that pregnant women in the Zhejiang province had insufficient iodine nutrition, which adversely affects the brain development of their fetus. 

During the period of sustainable elimination of IDD, China is considered to be a country that uses iodized table salt as its primary source of iodine [31,48,49]. A previous country-level study showed that iodized salt contributed to 83% of iodine nutrition source in the inland areas and nearly 70% in the coastal areas, though population living in coastal consumed more iodine-rich sea foods than people inland [49]. In this current study, the iodine content of edible salt was monitored in pregnant women at the household level. We observed that the participants living inland had adequate iodine intake, while those living in coastal had iodine deficiency. For those living in the coastal areas, participants consuming adequately iodized salt still had iodine deficiency with a slightly greater median UIC of 135.8 µg/L, compared with that in those consuming non-iodized salt (114 µg/L). In addition, more than 90% of pregnant women consumed adequately iodized salt at the province level, which reached the required standard of maintaining the sustainable elimination of IDD [8]. However, those consuming adequately iodized salt were marginally iodine-sufficient, and more than half of pregnant women in the province had iodine deficiency. These study results indicate that an increased coverage rate of adequately iodized salt in the pregnant population in Zhejiang would slightly enhance iodine intake; however, it may not be enough to correct iodine deficiency status to an optimal level in pregnancy. A slightly higher percentage of non-iodized salt was observed in pregnancy in coastal in the present study. Its reason may be related with local natural salt without adding iodine, which historically was produced from salt evaporation ponds and used in the coastal households in the Zhejiang province [50]. Therefore, efforts are needed to forbid non-iodized salt in the market. Besides USI policy needs to be strengthened and implemented, more efforts should be made to increase iodine intake in pregnancy, for example, increased iodine content standard in salt should be set only for pregnant woman population; a recommendation should be made for pregnant women to take an iodine-containing supplement. 

## 5. Conclusions

Our study showed that, overall, all pregnant women were iodine-deficient in the Zhejiang province. The pregnant population consistently consuming adequate iodized salt had marginally adequate iodine status. A single measure of increasing consumption rate of adequately iodized salt in pregnancy may slightly increase their iodine nutrition, but it may not correct iodine deficiency to an optimal level. More efforts are needed to increase iodine content in salt. Suggestions on treatment with iodine-containing supplements could be made to improve iodine nutrition in pregnancy.

## Figures and Tables

**Table 1:** Criteria for assessing iodine nutrition status in pregnancy based on median urinary iodine concentration (UIC).

Median UIC (μg/L)	Iodine Intake	Iodine Nutrition Status
<150	Insufficient	Iodine deficiency
150–249	Adequate	Optimal
250–499	Above requirements	-
≥500	Excessive	-

**Table 2:** Number of the total study population of pregnant women by maternal age and areas.

Variables	Number (%)
**Maternal Age**	
≤30 years	5973 (73.2)
>31 years	2186 (26.8)
**Areas**	
Coastal	4372 (53.6)
Inland	3787 (46.4)
Total	8159 (100)

**Table 3:** Median UIC in pregnancy by maternal age, consuming salt, and areas.

Variables	N	Median UIC, Interquartile Range (µg/L)	*p*-Value
**Maternal Age**			
≤30 years	6240	149.9 (93.3–212.3)	0.085
>31 years	2270	143.9 (92.5–204.3)	
**Salt**			
Non-iodized salt	450	99.6 (62.22–16.8)	<0.001
Adequately iodized salt	7392	151.9 (97.6–215.0)	
**Areas**			
Coastal	4075	130.1 (79.9–193.9)	<0.001
Inland	4435	158.1 (107.1–222.3)	

**Table 4:** Percentage of participants consuming salt by maternal age and areas.

Variables	Non-Iodized Salt	Adequately Iodized Salt	χ^2^ Value	*p*-Value
**Maternal Age**				
≤30 years	5.5 (314)	94.5 (5312)	3.006	0.083
>31 years	6.5 (136)	93.5 (1951)		
**Areas**				
Coastal	10.5(378)	89.5 (3216)	279.6	<0.001
Inland	1.7 (71)	98.3 (4147)		

**Table 5:** Percentage of UIC in pregnant women consuming different salt.

Salt	Percentage of UIC Levels, % (N)				Sum
	<150 µg/L	150–249 µg/L	250–499 µg/L	≥500 µg/L	
Non-iodized salt	71.6 (322)	16.7 (75)	7.5 (34)	4.2 (19)	100.0 (450)
Adequately iodized salt	49.5 (3662)	33.8 (2498)	13.3 (979)	3.4 (253)	100.0 (7392)
All salt	51.0 (4341)	32.9 (2803)	12.8 (1082)	3.3 (284)	100.0 (8510)

## Abbreviations

The following abbreviations are used in this manuscript:

USI	universal salt iodization
IDD	iodine deficiency disorder
UIC	urinary iodine concentration
WHO	World Health Organization
UNICEF	United Nations Children’s Fund
ICCIDD	International Council for the Control of Iodine Deficiency Disorders
SPSS	Statistical Package for the Social Sciences
